# Association between microRNA 671 polymorphisms and the susceptibility to soft tissue sarcomas in a Chinese population

**DOI:** 10.3389/fonc.2022.960269

**Published:** 2022-08-09

**Authors:** Peng Zhang, Xinling Li, Lingling Huang, Fulan Hu, Xiaoying Niu, Yang Sun, Weitao Yao

**Affiliations:** ^1^ Department of Bone and Soft Tissue Cancer, The Affiliated Cancer Hospital of Zhengzhou University, Henan Cancer Hospital, Zhengzhou, China; ^2^ College of Public Health, Zhengzhou University, Zhengzhou, China; ^3^ Department of Biostatistics and Epidemiology, School of Public Health, Shenzhen University Health Science Center, Shenzhen, China; ^4^ Department of Orthopaedic Oncology Surgery, Beijing Jishuitan Hospital, Peking University, Beijing, China

**Keywords:** miRNA-671, soft tissue sarcoma, rs1870238, rs2446065, gene polymorphisms

## Abstract

This study evaluated the association between the microRNA (miRNA) gene polymorphisms and the susceptibility to soft tissue sarcomas (STSs). In this case–control study, DNA was extracted from leukocytes in peripheral blood, which was collected from 169 STSs patients and 170 healthy controls. Three SNPs for miR-210, five SNPs for miR-206, two SNPs for miR-485, two SNPs for miR-34b, two SNPs for miR-671, and three SNPs for miR-381 were investigated and genotyped using a Sequenom Mass ARRAY matrix-assisted laser desorption/ionization-time of flight mass spectrometry platform. Unconditional logistic regression analysis was used to analyze the association between miRNA gene polymorphisms and the susceptibility to STSs. The results showed that miR-671 rs1870238 GC + CC (OR = 1.963, 95% CI = 1.258–3.064, *P* = 0.003) and miR-671 rs2446065 CG + GG (OR =1.838, 95% CI = 1.178–2.868, *P* = 0.007) may be genetic risk factors for STSs after adjustment for age and smoking. Therefore, this study suggests that individuals carrying the GC + CC genotype for miR-671 rs1870238 or the CG + GG genotype for miR-671 rs2446065 are susceptible to STSs.

## Introduction

Soft tissue sarcomas (STSs) are a highly heterogeneous group of malignant tumors arising from mesenchymal tissues. Histologically speaking, they are composed of many subtypes with ambiguous clinical and histopathological features, which lead to great challenges in their diagnosis and therapy (1). One important clinical challenge is the lack of useful biomarkers. The identification of biomarkers that can be used for primary prevention or to detect tumor responses to chemotherapy or radiotherapy may provide more effective clinical management approaches for clinicians. A growing amount of evidence suggests that miRNA dysregulation in STSs plays an important role in its progression and prognosis (2). The evidence of microRNA in STSs promotes the potential application of microRNA as a clinical biomarker, giving us one potential solution for preventing STSs.

MiRNAs are single-stranded non-coding RNAs that contain 18–25 nucleotides (3). MiRNAs can mediate gene expression by binding to target genes and inhibiting translation and protein synthesis at the post-transcriptional level (4). Most miRNAs are found within keygenomic regions thought to be involved in carcinogenesis. Many miRNAs have also been abnormally expressed in STSs, and the knowledge of miRNA expression patterns in STSs could identify specific signatures for the histological subtypes (5, 6). Furthermore, the different profiling of miRNA expression in tumor tissue of STSs compared with adjacent tissue may provide a clue for the diagnosis of STSs (7–9). Many studies recently have demonstrated that the altered expression of miRNAs is associated with the occurrence of numerous diseases, which may have a significant possibility of being used as biomarkers and targets of treatment for human illnesses. Structural genetic alterations, including chromosomal abnormalities (10), mutations (11), and single-nucleotide polymorphisms (SNPs), frequently occur in cancers and can affect miRNA expression (12).

SNPs represent an alternate nucleotide that occurs on average every 100 to 1,000 base pairs in vertebrates. Several studies have shown that SNP provided an approach for identifying possible genetic loci associated with diseases, including cancer susceptibility (13). The SNPs in miRNA genes (miRNA-SNP) may work in three possible ways: altering transcription of the primary miRNA transcript; the processing of the pri-miRNA and pre-miRNA; and through their effects on the modulation of miRNA–mRNA interactions (14). Furthermore, a single nucleotide change in a primary miRNA can greatly influence its stability and maturation or alter its activity. Therefore, miRNA-SNPs are associated with many types of cancer (15), including chronic lymphocytic leukemia, thyroid, gastric, and lung cancer. However, the association between polymorphism in the miRNA gene and the risk of STSs has not been fully studied.

Therefore, we first screened the miRNAs associated with the initiation and development of STSs in the literature, including miR-206 (16), miR-671, miR-381 (17), miR-210, miR-485 (18), and miR-34b (19). Then, we detected three SNPs (rs10902173, rs12364149, rs7935908) in miR-210, five SNPs (rs1537670, rs2397080, rs16882131, rs17578851, rs6920648) in miR-206, two SNPs (rs4143957, rs12886869) in miR-485, two SNPs (rs2187473, rs4938723) in miR-34b, two SNPs (rs1870238, rs2446065) in miR-671, and three SNPs (rs2281610, rs7149890, rs34038694) in miR-381, and investigated the susceptible genotype of STSs.

## Materials and methods

### Samples

A total of 169 patients with histologically diagnosed STSs were recruited from the Henan Province Cancer Hospital in Henan Province, China. There were 33 synovial sarcomas, 32 undifferentiated pleomorphic sarcomas, 31 fibrosarcomas, 21 liposarcomas, 16 leiomyosarcomas, 11 rhabdomyosarcomas, eight Ewing sarcomas of soft tissues, five malignant peripheral nerve sheath tumors, five spindle cell sarcomas, three alveolar soft part sarcomas, three angiosarcomas, and one clear cell sarcoma. Besides, 170 population-based controls were enrolled during the same period of time as the individuals for physical examinations without a previous history of cancer. Participants who smoked no less than one cigarette per day or those who smoked for more than half a year were categorized as “smokers”, and those who drank more than two times a week and who drank continuously for more than 6 months were categorized as “drinkers”. The standard of the criteria of “smoking” or “drinking”, the method of questionnaire and peripheral blood collection were all described in more detail in our previous study (20). The study was approved by the ethical committee of Zhengzhou University, and all the participating patients signed informed consent.

### Genomic DNA sample preparation from whole human blood

The extraction and evaluation of genomic DNA from leukocytes in peripheral blood were described in our previous study (20). Genomic DNA was extracted from whole blood, using the Blood DNA Kit (Bioteke Corporation, Beijing, China) according to the protocol of the manufacturer, and stored at −80 °C until use. The DNA purity and concentration were determined by spectrophotometric measurement of absorbance at 260 and 280 nm using a Thermo Scientific NanoDrop™ 8000 UV–Vis Spectrophotometer (Thermo Fisher Scientific Inc., Wilmington, DE, USA).

### Genotyping of polymorphic loci

Firstly, the miRNAs associated with STSs were screened in the literature, including miR-206 (16), miR-671, miR-381 (17), miR-210, miR-485 (18), and miR-34b (19). Next, we screened the functional region SNPs in the gene region, promoter proxy (TSS200), exon (missense and synonymous), and 3’ UTR region through the NCBI dbSNP database. Furthermore, we screened the validated and hot SNPs through the GWAS Catalog (https://www.ebi.ac.uk/gwas/), GWAS Atlas (https://atlas.ctglab.nl/), and GWAS Central (https://www.gwascentral.org/). Then identified with a cut-off value of *r^2^* = 0.8 and a minor allele frequency greater than 0.05 in the Chinese population by 1,000 Genomes. Finally, 21 SNPs, including functional region SNPs and validated and hot SNPs, were selected for genotyping. Because the detection frequency of the rs11606481 genotype was different from the CHB&CHS 1,000 Genomes database and the detection rate of rs4467881, rs11246190, and rs28524679 was less than 95%, we further analyzed the other 17 loci after removing the four loci.

The SNPs were genotyped using a SequenomMassARRAY^®^ matrix-assisted laser desorption/ionization-time of flight (MALDI-TOF) mass spectrometry platform (Sequenom Inc., San Diego, CA, USA). The genotyping experiment mainly included a PCR amplification reaction and a single-base extension reaction. Among these, PCR amplification is to obtain gene fragments containing SNP loci. The 5 µl reaction system was conducted, including 4 µl PCR master mix and 1 µl DNA (20 ng/µl). In the PCR amplification conditions, an initial pre-denaturation was performed at 94°C for 5 min, followed by 45 cycles at 94°C for 20 s, 56°C for 30 s, and 72°C for 60 s, and then a final exposure to 72°C for 3 min. In the single-base extended, 9 μl PCR reaction systems were conducted, including 2 µl EXTEND mix (AgenaBiocience, Inc.), and 7 µl SAP (AgenaBiocience, Inc.) + PCR reaction (product of the PCR amplification). In the single-base extended PCR conditions, an initial pre-denaturation was performed at 94°C for 30 s, followed by 40 cycles at 94 °C for 5 s, 52 °C for 5 s and 80°C for 5 s, 5 cycles at 52°C for 5 s, 80°C for 5 s, and then a final exposure to 72°C for 3 min.

**Figure 1:**
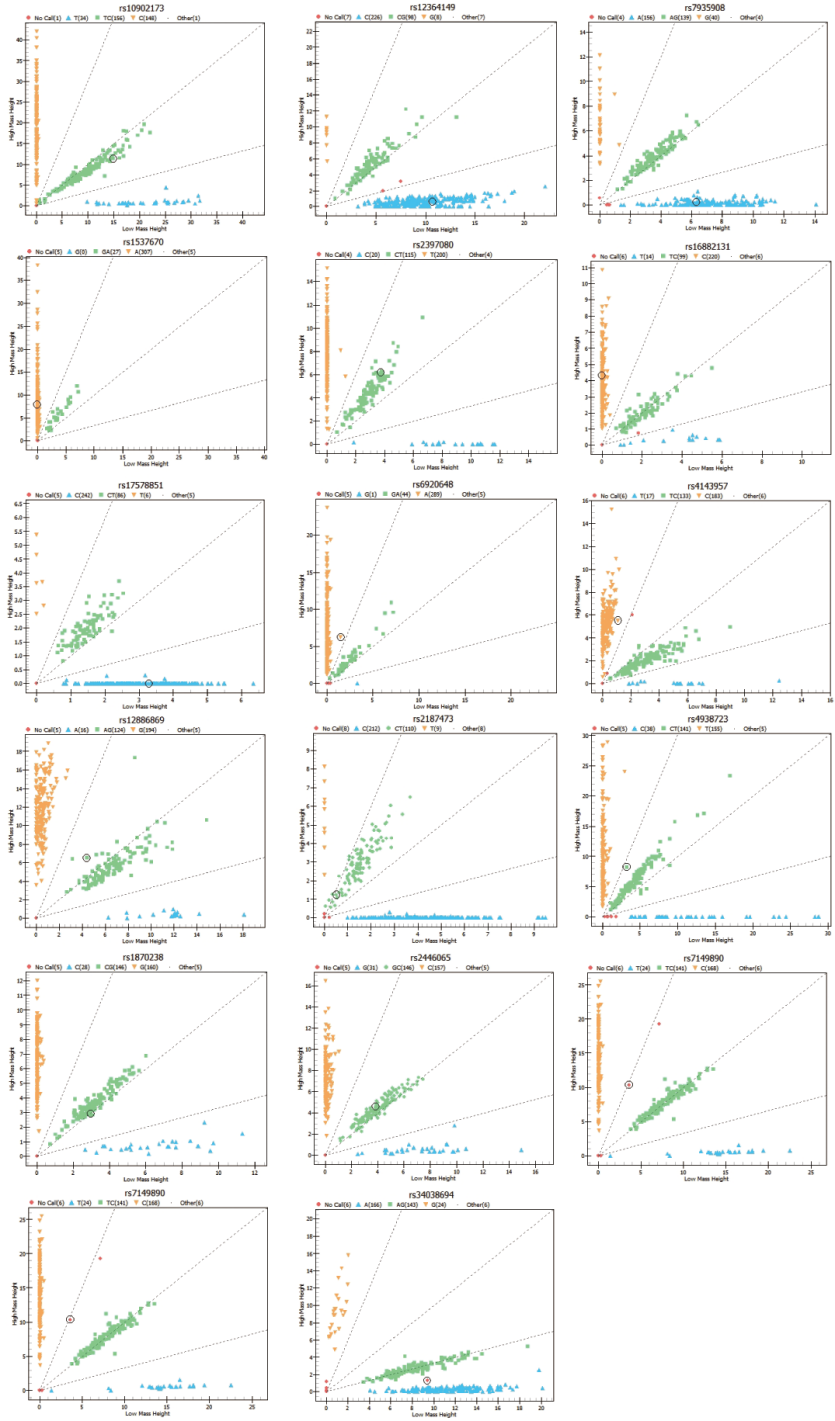
The genotype plots of miRNA polymorphisms. The X and Y axes represent the kurtosis of genotypes, respectively. The red square represents no call; the blue upright triangle, the green square, and the orange inverted triangle represent three different genotypes, respectively. In a good clustering plot, the two homozygous types are close to the horizontal axis and the vertical axis, while the heterozygous types are between them, showing a 45-degree angle. Clustering performance shows the evaluation of clustering efficiency, which ranges from 0 to 1.

The primers for the PCR reaction were designed using Assay Designer 3.1 software and synthesized by a biotechnology company. **Table 1** demonstrates the primer sequences of encoding miRNA genes polymorphic loci, and the genotype plots of seventeen SNPs are shown in **Figure 1**.

**Table 1:** Primers sequences for miRNA gene polymorphism.

Polymorphism		Primers sequences
miR-210rs10902173	Forward:	5’-ACGTTGGATGTCACAGGCACCTTTTCTCAG-3’
	Reverse:	5’-ACGTTGGATGAGCCTGGGTATTAGGATGTG-3’
miR-210 rs12364149	Forward:	5’-ACGTTGGATGTGATCCTCTGGGCACCTTC-3’
	Reverse:	5’-ACGTTGGATGTTGCTGACCCCTTGACCCTT-3’
miR-210 rs7935908	Forward:	5’-ACGTTGGATGATCCTCCAGCAGCCTGTCT-3’
	Reverse:	5’-ACGTTGGATGGACCCGGTCCTGATTTTAAC-3’
miR-206 rs1537670	Forward:	5’-ACGTTGGATGCCTTCCTCTGGTCATATTAC-3’
	Reverse:	5’-ACGTTGGATGACGCTTGCAATACACATGGC-3’
miR-206rs2397080	Forward:	5’-ACGTTGGATGAATCTTTCGGGCTGACCTTG-3’
	Reverse:	5’-ACGTTGGATGAGTGTTTTCAGAGCAGAAGC-3’
miR-206 rs16882131	Forward:	5’-ACGTTGGATGGCTGCACAAGAATAAGCCAG-3’
	Reverse:	5’-ACGTTGGATGTGCTTGGGACCAAATCCTTC-3’
miR-206 rs17578851	Forward:	5’-ACGTTGGATGAAGTGGAAAGGACAGCAGAG-3’
	Reverse:	5’-ACGTTGGATGGTGAGTGAGGTTCAGGAAAC-3’
miR-206 rs6920648	Forward:	5’-ACGTTGGATGAGCAGAAGCCCGACAAAAGG-3’
	Reverse:	5’-ACGTTGGATGTTTGGGTGCTTGTTGATGGG-3’
miR-485rs4143957	Forward:	5’-ACGTTGGATGTGTGACAAGTGGCTTCCCTC-3’
	Reverse:	5’-ACGTTGGATGCCCTGGAGTTGAAATTGTGG-3’
miR-485 rs12886869	Forward:	5’-ACGTTGGATGAGGTGCCCCTAGAGAAACTG-3’
	Reverse:	5’-ACGTTGGATGATAGAGAATCTACCCAGGGC-3’
miR-34b rs2187473	Forward:	5’-ACGTTGGATGGGTTTCCTCGCACTTGCAG-3’
	Reverse:	5’-ACGTTGGATGGAGAGAAGATGCCTGAGAAG-3’
miR-34b rs4938723	Forward:	5’-ACGTTGGATGTAGAAGGGAGGTCCTCAATG-3’
	Reverse:	5’-ACGTTGGATGGGATCTACTCAAGTCTCACC-3’
miR-671 rs1870238	Forward:	5’-ACGTTGGATGATCACTCCTCTGCCACCTTG-3’
	Reverse:	5’-ACGTTGGATGCCCTCCCCAGTTTCCAATG-3’
miR-671 rs2446065	Forward:	5’-ACGTTGGATGGGTGGAGTGTAGATGAAAAC-3’
	Reverse:	5’-ACGTTGGATGAGCTCAACAGCCTTTCTCTC-3’
miR-381 rs2281610	Forward:	5’-ACGTTGGATGTCCTAGAGATGACCAGATCC-3’
	Reverse:	5’-ACGTTGGATGTCCTTTGTCGCTAGAGTCTG-3’
miR-381 rs7149890	Forward:	5’-ACGTTGGATGTGGAGGTGGTATTGACCTTG-3’
	Reverse:	5’-ACGTTGGATGGAGCTGGATCATGAACACCC-3’
miR-381 rs34038694	Forward:	5’-ACGTTGGATGCCATAGGTCAGCTCTCCATC-3’
	Reverse:	5’-ACGTTGGATGGGAAAAGAGGCTGATTCTGG-3’

Forward represents the upstream primer and reverse represents the downstream primer.

### Statistical analysis

SPSS 21.0 software was used to analyze all the data (SPSS Inc., Chicago, IL, USA). The distribution of population characteristics and genotypes between two groups was analyzed using a chi-square test or *t*-test. A logistic regression model was used to calculate adjusted odds ratios (OR) and 95% confidence intervals (*CI*). The level of statistical significance was set at two-sided α = 0.05.

## Results

### General characteristics of subjects

The characteristics of the study population are presented in **Table 2**. In total, 170 controls and 169 cases were included in these analyses. The ages ranged from 18 to 85 years in cases, and the age in the cases (48.18 ± 15.16) was significantly younger than that in the controls (51.59 ± 11.08) (*P*= 0.019). The proportion of smokers in cases (16.6%) was less than that in controls (25.3%) (*P* = 0.048). The proportion of drinking status in cases (8.9%) was less than that in the controls (17.6%) (*P* = 0.017). There were no significant differences in gender between the two groups (*P* = 0.960).

**Table 2:** General Characteristics of theSubjects.

Characteristics	Controls (n=170)	Cases (n=169)	*χ^2^/t*	*P*
Gender				
Male	91 (53.5)	90 (53.3)	0.003	0.960
Female	79 (46.5)	79 (46.7)		
Age (years)	51.59±11.08	48.18±15.16	2.367	**0.019**
Smoking status				
No	127 (74.7)	141 (83.4)	3.897	**0.048**
Yes	43 (25.3)	28 (16.6)		
Drinking status				
No	140 (82.4)	154 (91.1)	5.664	**0.017**
Yes	30 (17.6)	15 (8.9)		

Bold values, X^2^:Chi-square test.

### Genotypic distribution of miRNA gene

The results of the Hardy–Weinberg balance shown in **Table 3** demonstrate that the genotype distribution for each genetic polymorphism locus did not deviate (*P >*0.05), and the allele frequencies were similar to those of Asians in the International Human Genome HapMap Project, suggesting that the controls had representativeness. As shown in **Table 4**, there was a statistically significant difference in miR-206 rs17578851, miR-671 rs1870238, and miR-671 rs2446065 between the STSs and controls (*P <*0.05). Besides, there were no significant differences in other genotypic frequencies between these two groups.

**Table 3:** Distribution of MiRNA Genotypic Frequencies inthecontrolgroup.

Gene	SNP	genotype	n	Allele Frequency	χ^2^/P
miR-210	rs10902173	TT	22	T 0.352	0.126/0.723
		TC	75	C 0.648	
		CC	72		
	rs12364149	CC	113	C 0.812	0.240/0.624
		CG	50	G 0.188	
		GG	7		
	rs7935908	GG	25	G 0.349	2.221/0.136
		GA	68	A 0.651	
		AA	76		
miR-206	rs1537670	AA	157	A 0.965	0.229/0.632
		AG	12	G 0.036	
		GG	0		
	rs2397080	TT	103	T 0.772	0.950/0.330
		TC	55	C 0.228	
		CC	11		
	rs16882131	TT	9	T 0.215	0.280/0.597
		TC	55	C 0.785	
		CC	106		
	rs17578851	CC	116	C 0.826	0.004/0.949
		CT	49	T 0.174	
		TT	5		
	rs6920648	AA	149	A 0.938	0.737/0.391
		AG	21	G 0.062	
		GG	0		
miR-485	rs4143957	CC	96	C 0.759	0.104/0.748
		CT	63	T 0.241	
		TT	9		
	rs12886869	GG	101	G 0.774	0.099/0.753
		GA	61	A 0.226	
		AA	8		
miR-34b	rs2187473	CC	111	C 0.821	3.272/0.070
		CT	57	T 0.179	
		TT	2		
	rs4938723	TT	78	T 0.662	1.481/0.224
		TC	69	C 0.338	
		CC	23		
miR-671	rs1870238	GG	95	G 0.747	0.003/0.960
		GC	64	C 0.253	
		CC	11		
	rs2446065	CC	92	C 0.732	0.103/0.748
		CG	65	G 0.268	
		GG	13		
miR-381	rs2281610	CC	4	C 0.167	0.137/0.711
		CT	48	T 0.833	
		TT	116		
	rs7149890	TT	`16	T 0.288	0.492/0.483
		TC	66	C 0.712	
		CC	88		
	rs34038694	AA	86	A 0.721	0.749/0.387
		AG	73	G 0.279	
		GG	11		

**Table 4:** The distribution of miRNA genetic polymorphism between the control group and the case group.

Polymorphisms	Controls (n=170)	Cases (n=169)	*X^2^*	*P*
miR-210rs10902173				
TT	22 (13.0)	12 (7.1)	3.280	0.194
TC	75 (44.4)	81 (47.9)		
CC	72 (42.6)	76 (45.0)		
T	119 (35.2)	105 (31.1)	1.309	0.253
C	219 (64.8)	233 (68.9)		
miR-210rs12364149				
CC	113 (66.5)	113 (69.8)	4.351	0.114
CG	50 (29.4)	48 (29.6)		
GG	7 (4.1)	1 (0.6)		
C	276 (81.2)	274 (84.6)	1.342	0.247
G	64 (18.8)	50 (15.4)		
miR-210rs7935908				
GG	25 (14.8)	15 (9.0)	2.641	0.267
GA	68 (40.2)	71 (42.8)		
AA	76 (45.0)	80 (48.2)		
G	118 (34.9)	101 (30.4)	1.534	0.215
A	220 (65.1)	231 (69.6)		
miR-206 rs1537670				
AA	157 (92.9)	150 (90.9)	0.445	0.505
AG	12 (7.1)	15 (9.1)		
GG	0 (0.0)	0 (0.0)		
A	326 (96.4)	315 (95.5)	0.426	0.514
G	12 (3.6)	15 (4.5)		
miR-206rs2397080				
TT	103 (60.9)	97 (58.4)	0.571	0.752
TC	55 (32.5)	60 (36.1)		
CC	11 (6.5)	9 (5.4)		
T	261 (77.2)	254 (76.5)	0.048	0.827
C	77 (22.8)	78 (23.5)		
miR-206rs16882131				
TT	9 (5.3)	5 (3.1)	2.510	0.285
TC	55 (32.4)	44 (27.0)		
CC	106 (62.4)	114 (69.9)		
T	73 (21.5)	54 (16.6)	2.596	0.107
C	267 (78.5)	272 (83.4)		
miR-206rs17578851				
CC	116 (68.2)	126 (76.8)	4.648	0.098
CT	49 (28.8)	37 (22.6)		
TT	5 (2.9)	1 (0.6)		
C	281 (82.6)	289 (88.1)	3.980	**0.046**
T	59 (17.4)	39 (11.9)		
miR-206rs6920648				
AA	149 (87.6)	140 (85.4)	1.264	0.532
AG	21 (12.4)	23 (14.0)		
GG	0 (0.0)	1 (0.6)		
A	319 (93.8)	303 (92.4)	0.544	0.461
G	21 (6.2)	25 (7.6)		
miR-485rs4143957				
CC	96 (57.1)	87 (52.7)	0.843	0.656
CT	63 (37.5)	70 (42.4)		
TT	9 (5.4)	8 (4.8)		
C	255 (75.9)	244 (73.9)	0.338	0.561
T	81 (24.1)	86 (26.1)		
miR-485rs12886869				
GG	101 (59.4)	93 (56.7)	0.254	0.881
GA	61 (35.9)	63 (38.4)		
AA	8(4.7)	8 (4.9)		
G	263 (77.354)	249 (75.9)	0.193	0.660
A	77 (22.6)	79 (24.1)		
miR-34b rs2187473				
CC	111 (65.3)	101 (62.7)	3.153	0.207
CT	57 (33.5)	53 (32.9)		
TT	2 (1.2)	7 (4.3)		
C	279 (82.1)	255 (79.2)	0.871	0.351
T	61 (17.9)	67 (20.8)		
miR-34b rs4938723				
TT	78 (45.9)	77 (47.0)	1.647	0.439
TC	69 (40.6)	72 (43.9)		
CC	23 (13.5)	15 (9.1)		
T	225 (66.2)	226 (68.9)	0.566	0.452
C	115 (33.8)	102 (31.1)		
miR-671rs1870238				
GG	95 (55.9)	65 (39.6)	9.025	**0.011**
GC	64 (37.6)	82 (50.0)		
CC	11 (6.5)	17 (10.4)		
G	254 (74.7)	212 (64.6)	8.028	**0.005**
C	86 (25.3)	116 (35.4)		
miR-671rs2446065				
CC	92 (54.1)	65 (39.6)	7.098	**0.029**
CG	65 (38.2)	81 (49.4)		
GG	13 (7.6)	18 (11.0)		
C	249 (73.2)	211 (64.3)	6.176	**0.013**
G	91 (26.8)	117(35.7)		
miR-381rs2281610				
CC	4 (2.4)	7 (4.3)	0.987	0.610
CT	48 (28.6)	44 (27.0)		
TT	116 (69.0)	112 (68.7)		
C	56 (16.7)	58 (17.8)	0.147	0.702
T	280 (83.3)	268 (82.2)		
miR-381rs7149890				
TT	`16 (9.4)	8 (4.9)	3.476	0.176
TC	66 (38.8)	75 (46.0)		
CC	88 (51.8)	80 (49.1)		
T	98 (28.8)	91 (27.9)	0.068	0.795
C	242 (71.182)	235 (72.1)		
miR-381rs34038694				
AA	86 (50.6)	80 (49.1)	0.299	0.861
AG	73 (42.9)	70 (42.9)		
GG	11 (6.5)	13 (8.0)		
A	245 (72.1)	230 (70.6)	0.185	0.667
G	95 (27.9)	96 (29.4)		

Bold values, X^2^: Chi-square test.

### miRNA genotype and risk of soft tissue sarcoma

The polymorphisms for miRNA and their ORs and 95% CI in STSs are shown in **Table 5**. Regarded wild homozygous as the reference group, miR-671 rs1870238GC+CC had a 1.929-fold (*OR* = 1.929, 95% CI = 1.248–2.982, *P* = 0.003) increased risk of STSs, and a 1.963-fold (*OR* =1.963, 95% CI = 1.258–3.064, *P* = 0.003) increased risk of STSs after adjusting for age, smoking status, and drinking status. The results also showed that miR-671 rs2446065 CG+GG had a 1.796-fold (*OR* = 1.796, 95% CI = 1.163–2.774, *P* = 0.008) increased risk of STSs and a 1.838-fold (*OR* = 1.838, 95% CI = 1.178–2.868, *P* = 0.007) increased risk of STSs after adjusting for age, smoking status, and drinking status. Moreover, there was no association between other locus polymorphisms for miRNA and the risk of STSs.

**Table 5:** The miRNA genetic polymorphisms and the risk of soft tissue sarcoma.

Polymorphisms	Controls (n=170)	Cases (n=169)	*OR (95%CI)^#^*	*P^#^*	OR (95%CI)^*^	P^*^
miR-210rs10902173						
TT	22 (13.0)	12 (7.1)	Ref		Ref	
TC	75 (44.4)	81 (47.9)	1.980 (0.916-4.278)	0.082	1.929 (0.881-4.224)	0.100
CC	72 (42.6)	76 (45.0)	1.935 (0.893-4.195)	0.094	2.015 (0.916-4.430)	0.081
TC+CC	147 (87.0)	157 (92.9)	1.958 (0.936-4.098)	0.075	1.970 (0.930-4.176)	0.077
miR-210rs12364149						
CC	113 (66.5)	113 (69.8)	Ref		Ref	
CG	50 (29.4)	48 (29.6)	0.960 (0.597-1.542)	0.866	0.921 (0.566-1.497)	0.739
GG	7 (4.1)	1 (0.6)	0.143 (0.017-1.180)	0.071	1.151 (0.018-1.265)	0.081
CG+GG	57 (33.5)	49 (30.2)	0.860 (0.541-1.365)	0.521	0.829 (0.516-1.331)	0.437
miR-210rs7935908						
GG	25 (14.8)	15 (9.0)	Ref		Ref	
GA	68 (40.2)	71 (42.8)	1.740 (0.846-3.580)	0.132	1.683 (0.808-3.508)	0.165
AA	76 (45.0)	80 (48.2)	1.754 (0.860-3.579)	0.122	1.860 (0.897-3.855)	0.095
GA+AA	144 (85.2)	151 (91.0)	1.748 (0.886-3.448)	0.107	1.772 (0.887-3.543)	0.105
miR-206 rs1537670						
AA	157 (92.9)	150 (90.9)	Ref		Ref	
AG	12 (7.1)	15 (9.1)	1.308 (0.593-2.887)	0.506	1.376 (0.604-3.137)	0.447
GG	0 (0.0)	0 (0.0)	-	-	-	-
AG+GG	12 (7.1)	15 (9.1)	1.308 (0.593-2.887)	0.506	1.376 (0.604-3.137)	0.447
miR-206rs2397080						
TT	103 (60.9)	97 (58.4)	Ref		Ref	
TC	55 (32.5)	60 (36.1)	1.158 (0.732-1.833)	0.530	1.042 (0.649-1.672)	0.864
CC	11 (6.5)	9 (5.4)	0.869 (0.345-2.188)	0.765	0.797 (0.310-2.051)	0.638
TC+CC	66 (39.1)	69 (41.6)	1.110 (0.717-1.718)	0.639	1.001 (0.638-1.570)	0.996
miR-206rs16882131						
TT	9 (5.3)	5 (3.1)	Ref		Ref	
TC	55 (32.4)	44 (27.0)	1.440 (0.450-4.607)	0.539	1.570 (0.478-5.156)	0.458
CC	106 (62.4)	114 (69.9)	1.936 (0.629-5.961)	0.250	2.065 (0.653-6.529)	0.217
TC+CC	161 (94.7)	158 (96.9)	1.766 (0.579-5.387)	0.317	1.896 (0.605-5.936)	0.272
miR-206rs17578851						
CC	116 (68.2)	126 (76.8)	Ref		Ref	
CT	49 (28.8)	37 (22.6)	0.695 (0.423-1.141)	0.151	0.715 (0.431-1.186)	0.193
TT	5 (2.9)	1 (0.6)	0.184 (0.021-1.599)	0.125	0.131 (0.015-1.168)	0.069
CT+TT	54 (31.8)	38 (23.2)	0.648 (0.399-1.053)	0.080	0.649 (0.396-1.065)	0.087
miR-206rs6920648						
AA	149 (87.6)	140 (85.4)	Ref		Ref	
AG	21 (12.4)	23 (14.0)	1.166 (0.618-2.200)	0.636	1.087 (0.567-2.083)	0802
GG	0 (0.0)	1 (0.6)	-	-	-	-
AG+GG	21 (12.4)	24 (14.6)	1.216 (0.648-2.283)	0.542	1.125 (0.590-2.145)	0.721
miR-485rs4143957						
CC	96 (57.1)	87 (52.7)	Ref		Ref	
CT	63 (37.5)	70 (42.4)	1.226 (0.784-1.918)	0.372	1.105 (0.698-1.748)	0.671
TT	9 (5.4)	8 (4.8)	0.981 (0.362-2.654)	0.970	1.065 (0.384-2.957)	0.904
CT+TT	72 (42.9)	78 (47.3)	1.195 (0.776-1.842)	0.418	1.100 (0.707-1.711)	0.673
miR-485rs12886869						
GG	101 (59.4)	93 (56.7)	Ref		Ref	
GA	61 (35.9)	63 (38.4)	1.122 (0.715-1.761)	0.618	1.005 (0.632-1.598)	0.982
AA	8 (4.7)	8 (4.9)	1.086 (0.392-3.011)	0.874	1.183 (0.414-3.380)	0.753
GA+AA	69 (40.6)	71 (43.3)	1.118 (0.723-1.726)	0.617	1.024 (0.656-1.601)	0.916
miR-34b rs2187473						
CC	111 (65.3)	101 (62.7)	Ref		Ref	
CT	57 (33.5)	53 (32.9)	1.022 (0.644-1.620)	0.927	1.005 (0.627-1.612)	0.983
TT	2 (1.2)	7 (4.3)	3.847 (0.781-18.946)	0.098	3.828 (0.761-19.254)	0.103
CT+TT	59 (34.7)	60 (37.3)	1.118 (0.713-1.751)	0.627	1.103 (0.697-1.747)	0.675
miR-34b rs4938723						
TT	78 (45.9)	77 (47.0)	Ref		Ref	
TC	69 (40.6)	72 (43.9)	1.057 (0.670-1.668)	0.812	0.965 (0.604-1.541)	0.880
CC	23 (13.5)	15 (9.1)	0.661 (0.321-1.361)	0.261	0.649 (0.310-1.361)	0.252
TC+CC	92 (54.1)	87 (53.0)	0.958 (0.623-1.473)	0.845	0.887 (0.570-1.380)	0.595
miR-671rs1870238						
GG	95 (55.9)	65 (39.6)	Ref		Ref	
GC	64 (37.6)	82 (50.0)	1.873 (1.189-2.950)	**0.007**	1.915 (1.203-3.049)	**0.006**
CC	11 (6.5)	17 (10.4)	2.259 (0.993-5.136)	**0.052**	2.232 (0.971-5.135)	**0.059**
GC+CC	75 (44.1)	99 (60.4)	1.929 (1.248-2.982)	**0.003**	1.963 (1.258-3.064)	**0.003**
miR-671rs2446065						
CC	92 (54.1)	65 (39.6)	Ref		Ref	
CG	65 (38.2)	81 (49.4)	1.764 (1.119-2.781)	**0.015**	1.813 (1.137-2.889)	**0.012**
GG	13 (7.6)	18 (11.0)	1.960 (0.898-4.279)	0.091	1.960 (0.887-4.333)	0.096
CG+GG	78 (45.9)	99 (60.4)	1.796 (1.163-2.774)	**0.008**	1.838 (1.178-2.868)	**0.007**
miR-381rs2281610						
CC	4 (2.4)	7 (4.3)	Ref		Ref	
CT	48 (28.6)	44 (27.0)	0.524 (0.144-1.912)	0.328	0.535 (0.142-2.010)	0.354
TT	116 (69.0)	112 (68.7)	0.552 (0.157-1.937)	0.353	0.629 (0.174-2.275)	0.480
CT+TT	164 (97.6)	156 (95.7)	0.544 (0.156-1.893)	0.338	0.600 (0.168-2.147)	0.432
miR-381rs7149890						
TT	`16 (9.4)	8 (4.9)	Ref		Ref	
TC	66 (38.8)	75 (46.0)	2.273 (0.914-5.651)	0.077	2.274 (0.902-5.735)	0.082
CC	88 (51.8)	80 (49.1)	1.818 (0.738-4.477)	0.193	2.070 (0.828-5.179)	0.120
TC+CC	154 (90.6)	155 (95.1)	2.013 (0.837-4.841)	0.118	2.163 (0.887-5.274)	0.090
miR-381rs34038694						
AA	86 (50.6)	80 (49.1)	Ref		Ref	
AG	73 (42.9)	70 (42.9)	1.031 (0.659-1.612)	0.894	0.887 (0.557-1.412)	0.612
GG	11 (6.5)	13 (8.0)	1.270 (0.538-2.998)	0.585	1.297 (0.538-3.128)	0.562
AG+GG	84 (49.4)	83 (50.9)	1.062 (0.691-1.633)	0.783	0.939 (0.602-1.464)	0.781

P^#^A logistic regression model was used to analyze the association between polymorphism in miRNA with soft tissue sarcoma risk.

P^*^A logistic regression model was used to analyze the association between polymorphism in miRNA with soft tissue sarcoma riskadjusted for age,smokingstatus, and drinkingstatus.

Ref: The reference group when comparing.

Bold values, X^2^:Chi-square test. OR, odds ratio; CI, confidence interval.

## Discussion

This hospital-based case–control study showed a significant association between the miR-671 polymorphism (rs1870238 and rs2446065) and STS risk in humans from Henan Province, China. Individuals carrying the heterozygous GC genotype or with the homozygous CC genotype in rs1870238 had a 1.929-fold increased risk of developing STSs compared with individuals with the GG wild-type. Individuals carrying the heterozygous CG genotype or with the homozygous GG genotype in rs1870238 had a 1.796-fold increased risk of developing STSs compared with individuals with the CC wild-type.

STSs include more than 70 histological subtypes that may occur at any age. Among these different heterogeneous subtypes of STSs, aggressive high-grade malignancies often arise in adolescents and young adults, such as rhabdomyosarcoma, synovial sarcoma, Ewing sarcoma, and osteosarcoma (21).In this study, the age of the cases ranged from 18 to 85 years old, and the age of the cases (48.18 ± 15.16) was lower than the median diagnosis age of STSs, which is generally 60 years. We speculated that the difference between the actual mean age in the cases and the mean age at diagnosis of STSs may be due to the Berkson bias and lots of histological subtypes. Recently, a study also indicated that the peak or average age of STS onset varies with different histological subtypes. For example, Aaron et al. (2021) reported that synovial sarcoma presents at a younger mean age of 39 years at diagnosis (22). This study case had numerous synovial sarcomas, which may decrease the age of cases. Besides, a case–control study indicated that smoking and alcohol were potential risk factors for sarcomas (23). Therefore, we further analyzed the association between polymorphism in the miRNA gene and the susceptibility of STSs adjusted for age, smoking status, and drinking status and found that miR-671rs1870238GC+CC and miR-671 rs2446065 CG+GG may be risk factors for STSs (*OR* = 1.963, 95% CI = 1.258–3.064, *P* = 0.003 and *OR* = 1.838, 95% CI = 1.178–2.868, *P* = 0.007, respectively).

MiR-671, located at 7q36.1, serves as a suppressor or an oncogene in different tumors and plays a vital role in the biological process of many types of cancer, particularly in osteosarcoma (24), breast cancer (25), and non-small cell lung cancer (26). MiR-671 could directly target microfibril-associated glycoproteins (26), tripartite motif 14 (27), forkhead box protein P2 (28) and M1 (25), TNF receptor-associated factor 3 (29), and cyclin D2 (30), involved in the proliferation, migration, and invasion of tumor cells. Besides, miR-671 may also regulate the PI3k/Akt signaling (31) and the Wnt signaling pathways (32) that are closely related to the occurrence and development of cancers. miR-671 plays a crucial role in the oncogenesis of tumors by silencing *SMARCB1* which manifests frequently with a loss-of-function mutation in malignant neoplasms (33). Furthermore, in previous studies, overexpression of miR-671 was found in epithelioid sarcomas (34), malignant peripheral nerve sheath tumors, and synovial sarcomas (17), implicating it as a promising therapeutic target for sarcomas. However, the association between genetic polymorphisms of miR-671 and the risk of STSs is ill-defined. In this study, we first identified that rs1870238 and rs2446065 of miR-671 were found to be associated with STS risk, which raised the most concerns and warranted further study. This evidence promotes the potential application of miR-671 polymorphisms as a clinical biomarker, giving us new hope for the early prevention and diagnosis of STSs.

MiR-206, located at 6p12.2, belongs to one of the muscle-specific miRNAs. MiR-206 could suppress myogenic differentiation and muscle cell proliferation through inhibition of DNA synthesis  (35). Therefore, an inhibitory effect on cell growth can be observed following a forced expression of miR-206 in rhabdomyosarcoma, which is one kind of myogenic sarcoma, both *in vitro* and *in vivo* (36, 37). MiR-206, highly expressed in normal skeletal muscle, was downregulated in leiomyosarcoma, normal smooth muscle (38), and rhabdomyosarcoma (37, 39). In particular, lowmiR-206 expression correlated with poor overall survival in metastatic embryonal rhabdomyosarcoma and alveolar rhabdomyosarcoma cases without PAX3/7–FOXO1 fusion genes (40). However, MiR-206 was found to be overexpressed in epithelioid sarcomas (34) and sera of rhabdomyosarcoma patients (41). One possible reason for the controversial result is that the reference person or tissue was different in the above studies. Muscle-specific miRNA levels were usually lower in rhabdomyosarcoma compared with skeletal muscle but generally higher than in other normal tissues. A study provided evidence that miR-206 rs6920648 (HR = 0.77 (95% CI = 0.61–0.97, p-value = 0.02) was associated with breast cancer survival (42). But there is no study on the association of miR-206 gene polymorphisms with STS risk. In this study, the results showed the loci distribution of miR-206 rs17578851 between the cases and controls was a significant difference (*P <*0.05) through the chi-square test. However, there was no association between miR-206 rs17578851 and STS risk with logistic regression analysis.

Both miR-210 and miR-34b are located on chromosomal 11. MiR-210 is a well-known responder to hypoxia, which is expressed in a wide range of cells and involves numerous biological processes (43). A previous study showed that miR-210 could directly regulate HIF3α in hypoxia-responsive STSs (18). Besides, miR-210 was upregulated in malignant peripheral nerve sheath tumors compared with neurofibromas (44). Furthermore, hypoxia-induced miR-210 promoter demethylation enhances proliferation, autophagy, and angiogenesis of schwannoma cells (45). MiR-210 was also found to decrease expression in angiosarcoma cells both *in vivo* and *in vitro*, and was associated with angiosarcoma cell proliferation by target E2F3 and ephrin A3 (46). The expression levels of miR-210 were correlated with the prognosis and age of tumor onset in a gender-specific manner in STS patients (47). MiR-34b was silenced in numerous cancers by DNA methylation of its promoter (48) and became an important tumor suppressor in many sarcoma types. For instance, miR-34b is downregulated in synovial Sarcoma relative to other sarcomas (5), the methylation levels of miR-34b were associated with STSs clinical stage (19), and miR-34a levels inversely correlate with poor patient survival outcomes indicating its potential role as a diagnostic marker in Ewing sarcoma (49). However, a recent study showed that miR-34b expression levels were significantly higher in Ewing’s sarcoma tumors compared to normal tissue and acted as a tumor oncogene, promoting Ewing’s sarcoma cell proliferation, migration, and invasion by downregulating Notch1 (50). Furthermore, miR-34b polymorphisms have provided evidence of association with cancer risk and survival. For example, Jeannette et al. (2013) found that miR-34b rs4938723 was associated with breast cancer survival (42). Qi et al. (2014) suggested that miR-34b rs4938723 was a susceptible locus for hepatocellular cancer and colorectal cancer (51) and many other studies have also shown that miR-34b rs4938723 variant may be a disk factor for the development of prostate cancer (52) and acute lymphoblastic leukemia (53). However, there was no study on the association of miR-210 and miR-34b polymorphisms with STS risk. Our study did not find an association between miR-210 and miR-34b polymorphisms and the risk of STSs either.

Both miR-485 and miR-381 are located on chromosomal 14. Little is known about the relationship between miR-485 and miR-381 and STSs. In previous studies, miR-485 was reported to be decreased in osteosarcoma cells, and overexpression of miR-485-3p restrained osteosarcoma cell proliferation, migration, and sphere formation (54). Besides, miR-485 could indirectly regulate HIF3α in hypoxia-responsive STSs (18). MiR-485 has also been found to be associated with drug-resistant rhabdomyosarcoma (55). As for miR-381, one study reported that miR-381 overexpression in epithelioid sarcomas (34). There was no study on the association of miR-485 and miR-381 polymorphisms with STS risk. Our study did not find an association between miR-485 and miR-381 polymorphisms and STS risk either.

However, there are still some limitations to our study. Firstly, miRNA-SNPs in each pathological classification of STSs were not analyzed due to the limited sample of different pathological types. Secondly, further research is needed to elucidate the target gene and mechanism of miR-671 rs1870238 and rs1870238 in STSs. Thirdly, there are racial differences in gene polymorphism, and the results need to be further verified in other populations.

## Conclusion

miR-671 rs1870238 GC+CC and miR-671 rs2446065 CG+GG may be genetic risk factors for STSs, suggesting that individuals carrying the GC+CC genotype for miR-671 rs1870238 or the CG+GG genotype for miR-671 rs2446065 are susceptible to STSs.

## Data availability statement

The raw data supporting the conclusions of this article will be made available by the authors, without undue reservation.

## Ethics statement

This study was reviewed and approved by the Ethical Committee of Zhengzhou University. The patients/participants provided their written informed consent to participate in this study.

## Author contributions

PZ designed this research and wrote this paper. XL and FH analyzed data. LH and XN collected the blood samples. YS and WY instructed this study. All authors listed have made a substantial, direct, and intellectual contribution to the work and approved it for publication.

## Funding

This study was financially supported by the grant of Scientific and Technological Innovation Outstanding Young Talent Training Project from the Health Commission of Henan Province (No. YXKC2021031).

## Acknowledgments

All the authors thank all the subjects who voluntarily joined this study.

## Conflict of interest

The authors declare that the research was conducted in the absence of any commercial or financial relationships that could be construed as a potential conflict of interest.

## Publisher’s note

All claims expressed in this article are solely those of the authors and do not necessarily represent those of their affiliated organizations, or those of the publisher, the editors and the reviewers. Any product that may be evaluated in this article, or claim that may be made by its manufacturer, is not guaranteed or endorsed by the publisher.
